# Fabrication of Bifacial-Modified Perovskites for Efficient Semitransparent Solar Cells with High Average Visible Transmittance

**DOI:** 10.3390/molecules30061237

**Published:** 2025-03-10

**Authors:** Dazheng Chen, Wenjing Shi, Yan Gao, Sai Wang, Baichuan Tian, Zhizhe Wang, Weidong Zhu, Long Zhou, He Xi, Hang Dong, Wenming Chai, Chunfu Zhang, Jincheng Zhang, Yue Hao

**Affiliations:** 1 State Key Laboratory of Wide Bandgap Semiconductor Devices and Integrated Technology, Faculty of Integrated Circuit, Xidian University, Xi’an 710071, China; 2 Science and Technology on Reliability Physics and Application of Electronic Component Laboratory, China Electronic Product Reliability and Environmental Testing Research Institute, Guangzhou 511370, China

**Keywords:** semitransparent perovskite solar cells, average visible transmittance, bifacial modification, buried interlayer, surface passivation

## Abstract

Semitransparent perovskite solar cells (PSCs) that possess a high-power conversion efficiency (PCE) and high average visible light transmittance (AVT) can be employed in applications such as photovoltaic windows. In this study, a bifacial modification comprising a buried layer of [4-(3,6-Dimethyl-9H-carbazol-9-yl) butyl] phosphonic acid (Me-4PACz) and a surface passivator of 2-(2-Thienyl) ethylamine hydroiodide (2-TEAI) was proposed to enhance device performance. When the concentrations of Me-4PACz and 2-TEAI were 0.3 mg/mL and 3 mg/mL, opaque PSCs with a 1.57 eV perovskite absorber achieved a PCE of 22.62% (with a V_OC_ of 1.18 V) and retained 88% of their original value after being stored in air for 1000 h. By substituting a metal electrode with an indium zinc oxide electrode, the resulting semitransparent PSCs showed a PCE of over 20% and an AVT of 9.45%. It was, therefore, suggested that the synergistic effect of Me-4PACz and 2-TEAI improved the crystal quality of perovskites and the carrier transport in devices. When employing an absorber with a wider bandgap (1.67 eV), the corresponding PSC obtained a higher AVT of 20.71% and maintained a PCE of 18.73%; these values show that a superior overall performance is observed compared to that in similar studies. This work is conductive to the future application of semitransparent PSCs.

## 1. Introduction

Photovoltaic technologies can convert clean solar energy to electricity, which can benefit our future energy supply. In addition to large-scale power plants, building-integrated photovoltaics (BIPV) can be utilized in the application of PV windows, roofs, walls, and railings [1]. Modern skyscrapers now increasingly use glass in facades, while semitransparent solar cells with a high-power conversion efficiency (PCE) and high average visible light transmittance (AVT; over 20%) [2] are promoting the development of power glass and sustainable buildings. Among the existing PV technologies, perovskite solar cells (PSCs) have attracted significant attention because of their adjustable bandgap and color, high PCE, low cost, and flexibility [3,4,5]. Semitransparent perovskite solar cells (PSCs) can be constructed by substituting the opaque metal electrode, but the primary challenge associated with balancing the power conversion efficiency (PCE) and optical transmittance lies in the development of high-quality perovskite films and minimizing the energy lost in devices.

To achieve high-performance levels, most research has focused on the hole transport layer (HTL)/perovskite/electron transport layer (ETL) in p-i-n PSCs, including modifications to the solvent [6], interface [7], passivation [8], component [9], and energy-band [10]. For PSCs with a typical ITO/NiO_x_/perovskite/fullerene/Ag structure, the relatively poor contact at the interface of NiO_x_/perovskite reduces the carrier transfer and limits the growth of the perovskite, which can be improved by inserting a buried layer of inorganic or organic salts [11,12] and organic self-assembled monolayers (SAMs, Me-4PACz, MeO-2PACz) [13,14,15]. Precursor engineering can not only change the bandgap and color of perovskites [16] but also obtain the optimal components required for controllable and scalable crystallization in perovskites [17,18,19]. In recent years, passivation strategies have been used in the molecular layer of ammonium cations and two-dimensional perovskite to passivate the defects in the bulk and/or surface perovskites (uncoordinated PbI_2_, A^+^ and X^−^ vacancies) and minimize the energy loss associated with non-radiative recombination [20,21,22,23], including PEAI, BAI, 2-TEAI, PEABr, and TABr. By combining these methods, the PCEs of opaque PSCs have exceeded 26%, thus achieving superior stability under environmental stress [24]. Regarding semitransparent PSCs, most investigations focus on transparent electrodes, optimizing the perovskite thickness, and the outer photonic crystals [25,26,27,28,29]. Most devices with an AVT over 30% show a PCE lower than 10%, while most devices with an AVT of ~20% achieve a PCE of approximately 14%; meanwhile, PSCs with an efficiency over 20% rarely achieve an AVT over 10%. Therefore, simultaneously achieving a high PCE and high AVT remains a challenge.

In this work, we propose a bifacial modification of perovskite to enhance the device performance of opaque and/or semitransparent PSCs. This strategy is represented by the synergistic effect of a Me-4PACz buried layer and a 2-TEAI surface passivator. Attributed to improvements in the quality and hydrophobicity of the perovskite, the alignment of the energy level at the perovskite/electrode interface, and the loss of carrier transport and non-radiative recombination, the p-i-n opaque 1.57 eV-PSCs led to a PCE of 22.62% (steady stable PCE of 21.77%) and a V_OC_ of 1.18 V. They also led to good stability and were demonstrated to be repeatable. The semitransparent PSCs showed a PCE of over 20% and an AVT of 9.45%. In addition, the AVT of the device with a wider bandgap absorber (1.67 eV) increased to 20.71%, and the PCE was maintained at 18.73%. In this work, we created semitransparent PSCs that exhibited a more balanced performance (PCE and AVT) that can be employed in BIPV-related areas in the future.

## 2. Results and Discussion

**Figure 1:**
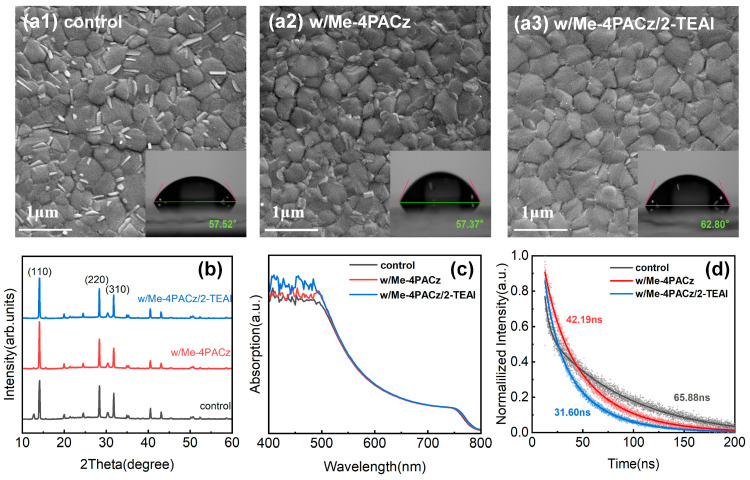
(**a1**–**a3**) SEM and water contact angle images, (**b**) XRD patterns, (**c**) light absorption spectra, and (**d**) TRPL spectra of the NiO_x_/perovskite, NiO_x_/Me-4PACz/perovskite, and NiO_x_/Me-4PACz/perovskite/2-TEAI test samples.

**Figure 2:**
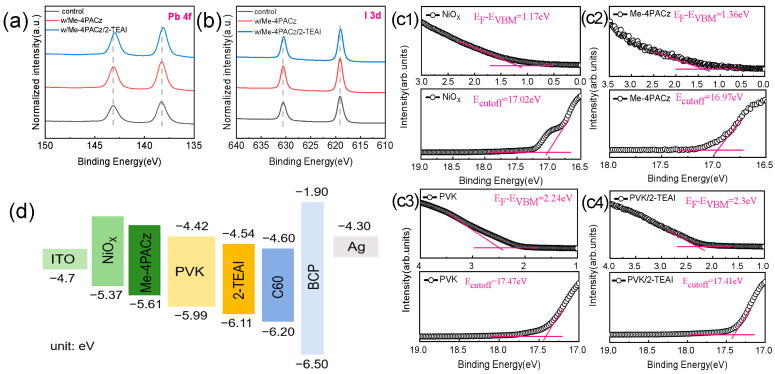
(**a**) Pb 4f and (**b**) I 3d XPS spectra; (**c1**–**c4**) UPS spectra of NiO_x_, Me-4PACz, and perovskite films with and without 2-TEAI modification; and (**d**) energy level diagram of PSCs.

Organic–inorganic hybrid perovskites comprising MA_0.72_FA_0.28_Pb(I_0.85_Cl_0.15_)_3_ were chosen as the photo-active layer of p-i-n PSCs; then, opaque devices were fabricated and investigated before semitransparent devices were created. Here, the bifacial modification of perovskites was performed by introducing a buried self-assembled monolayer (SAM) of Me-4PACz and a surface passivator of 2-TEAI. Their effect on the microstructure and physical properties of perovskites was as follows: As shown in the inserts of Figure 1, compared with NiO_x_ (with or without Me-4PACz)/perovskites, the water contact angle of perovskites increased from ~57° to 62.8° when the 2-TEAI treatment was introduced; this more hydrophobic surface is able to improve the stability of perovskite film and PSCs. The microscopic SEM images and corresponding XRD patterns are shown in Figure 1a1–a3,b. The XRD diffraction peaks at 13.70°, 27.96°, and 31.37° were assigned to the (110), (220), and (310) planes of perovskites, respectively. Meanwhile, there were many tiny rods on the surface of the NiO_x_/perovskite (control sample), which may be demonstrated to be excess PbI_2_ by the XRD diffraction peak at ~12.4° [29]. This issue was overcome by inserting Me-4PACz and completely resolved after introducing the 2-TEAI passivator, as shown in Figure 1b. It is suggested that the buried SAM of Me-4PACz addressed the unsatisfactory interfacial contact between perovskite and NiO_x_, which could improve the perovskite crystallinity and interfacial charge transfer [14]. However, the inadequate effect of the SAMs on the defect passivator could be further compensated by the spin-coating of 2-TEAI on perovskite; this cooperation mechanism is discussed in detail in the next section. The light absorptions of the ITO/NiO_X_/perovskite (control), ITO/NiO_X_/Me-4PACz/perovskite (w/Me-4PACz), and ITO/NiO_X_/Me-4PACz/perovskite/2-TEAI (w/Me-4PACz/2-TEAI) samples are shown in Figure 1c. It can be seen that the light absorption capacity of perovskite when the wavelength ranged from 400 nm to 500 nm was slightly improved by the bifacial modification method without a change in the optical bandgap (see Figure S1); this improved the photocurrent generated in PSCs. In addition, it can be seen in Figure 1d that the optimized perovskite sample of ITO/NiO_x_/Me-4PACz/perovskite/2-TEAI obtained the shortest carrier lifetime of 31.6 ns (Table S1). This means that it also performed the most efficient hole extraction. This is supported by the XPS core-level spectra shown in Figure 2 and Figure S2 (Supplementary Materials). The appearance of the S 2p peak demonstrated that the 2-TEAI was located on the (near) surface of the perovskite film (Figure S2). After passivation, the Pb 4f, I 3d, and N 1s peaks shifted to lower binding energies, which could be attributed to ionic bonds (2-TEA^+^ with PbI_6_^4−^), hydrogen bonds (2-TEA^+^ with I^−^), and/or coordination of S and Pb^2+^. This result agreed well with the findings of [30]. All these evolutions of perovskite films can enable the fabrication of high-performance and stable PSCs.

To explore the possible effects of bifacial modification on carrier transport, ultraviolet photoelectron spectroscopy (UPS) tests were performed. The electronic energy levels of the NiO_x_, Me-4PACz, perovskite, and perovskite/2-TEAI samples are shown in Figure 2c1–c4,d. According to the cutoff binding energy (E_cutoff_), the differences between the Femi levels (E_F_), the valence band maximum (E_VBM_), and the reported calculation method [31], their work functions (WFs) were 4.2 eV, 4.25 eV, 3.75 eV, and 3.81 eV, respectively; in addition, the calculated E_VBM_ values were −5.37 eV, −5.61 eV, −5.99 eV, and −6.11 eV, respectively. The energy levels of the minimum conduction band (E_CBM_) for perovskite and modified perovskite were −4.42 eV and −4.54 eV, respectively, when the bandgap was set to 1.57 eV. As is also clear in Figure 2d, the introduction of Me-4PACz and 2-TEAI improved the energy level alignments at both the hole-transport buried interface of perovskite and the electron-transport upper surface of perovskite. This enabled the enhancement of carrier extraction and collection near the anode and cathode. Therefore, the bifacial modification strategy can not only improve the quality of perovskite film but also strengthen the ability of perovskite-related interfaces to perform carrier transport; this may lead to the fabrication of PSCs with an efficient photovoltaic performance.

**Figure 3:**
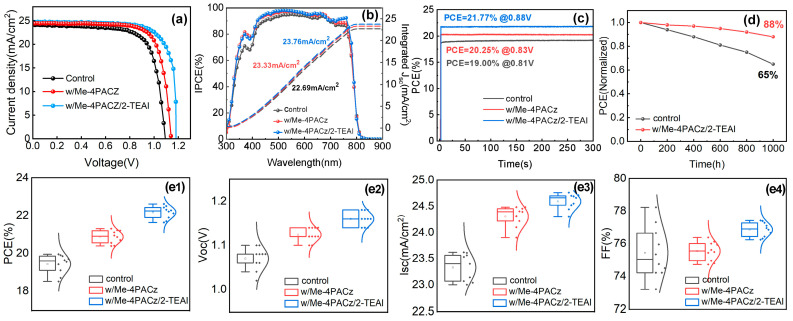
(**a**) JV curves; (**b**) EQE spectra; (**c**) steady-state PCEs; (**d**) storage stability; and (**e1**–**e4**) statistical photovoltaic parameters of the control, Me-4PACz-modified, Me-4PACz, and 2-TEAI-modified PSCs.

The opaque devices comprising ITO/NiO_x_/Me-4PACz/MA_0.72_FA_0.28_Pb(I_0.85_Cl_0.15_)_3_/2-TEAI/C_60_/BCP/Ag, as well as the control devices, were tested under AM 1.5G conditions, and the results are displayed in Figure 3 and Table 1. The unmodified control device exhibited a PCE of 19.23%, a V_OC_ of 1.08 V, a J_SC_ of 24.16 mA/cm^2^, and an FF of 73.68%. After inserting 0.3 mg/mL of Me-4PACz, the PCE increased to 21.31%, with a V_OC_ of 1.14 V, a J_SC_ of 24.48 mA/cm, and an FF of 76.38%. In addition, after 2-TEAI (3.0 mg/mL) passivation, the PCE reached 22.62%, with overall improvements in the V_OC_ (1.18 V), J_SC_ (24.76 mA/cm^2^), and FF (77.43%). The optimization of the concentration of Me-4PACz and 2-TEAI can be found in the supporting information (Figure S3 and Table S2). The integrated current density values for the corresponding device in the EQE spectra (Figure 3b) match the J_SC_ in the JV curves, which verifies the accuracy of the JV tests. In Figure 3c, the control and single and bifacial-modified PSCs achieved steady-state PCEs of 19%, 20.25%, and 21.77%, respectively, under their maximum power output points. This enhanced photovoltaic performance is confirmed by the statistical results shown in Figure 3e1–e4. The unencapsulated PSCs were evaluated further in environmental stability tests. When the bifacial-modified device was stored in a laboratory atmosphere for 1000 h, the PCE remained at 88% of its original value (Figure 3d). After being stored in a N_2_-filled glovebox for 1000 h, the PCE of the PSCs remained at 92% of its original value (Figure S4a). For the thermal stability analysis, the PSC was heated at 100 °C in air for 5 h, and the PCE remained at 79% of its original value (Figure S4b). The stability of the target PSCs was superior to that of the control device in the same conditions.

**Figure 4:**
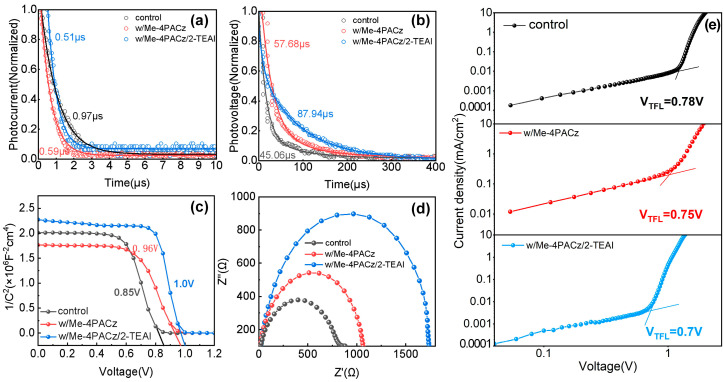
(**a**) TPC; (**b**) TPV; (**c**) M-S; (**d**) Nyquist curves; and (**e**) space-charge-limited current (SCLC) results for the control, Me-4PACz-modified, Me-4PACz and 2-TEAI-modified PSCs.

The carrier dynamics in PSCs were investigated to understand their photovoltaic performance. Transient photocurrent (TPC) and photovoltage (TPV) tests were performed to analyze the carrier transport and recombination in the PSCs. It is clear in Figure 4a that the devices tended to decay more rapidly after modification and that the current decayed faster in the bifacial-modified PSC (0.51 μs); this, therefore, represents the most efficient carrier extraction process. In Figure 4b, the longest recombination lifetime of the bifacial-modified PSC, at 87.94 μs, suggests that carrier recombination was suppressed satisfactorily. In addition, the highest built-in voltage (V_bi_) of 1.0 V during the Mott–Schottky (M-S) test coincided with the higher V_OC_, indicating a lower loss of non-radiative recombination (see Figure 4c). This was further confirmed by the large-radius semicircles or larger recombination resistance (R_rec_) present in the Nyquist plots shown in Figure 4d; the fitted values can be found in Table S3. In addition, the lowest trap-filled limit voltage (V_TFL_, 0.7 V) and corresponding trap density of 1.2 × 10^15^ cm^−3^ (Table S4), which was calculated from the SCLC tests (Figure 4e), made the efficient carrier transport possible. As a result, the bifacial modification of perovskite by Me-4PACz and 2-TEAI could lead to opaque PSCs with a higher performance; this provides a basis for fabrication of semitransparent devices with a high PCE and high average visible transmittance (AVT).

**Figure 5:**
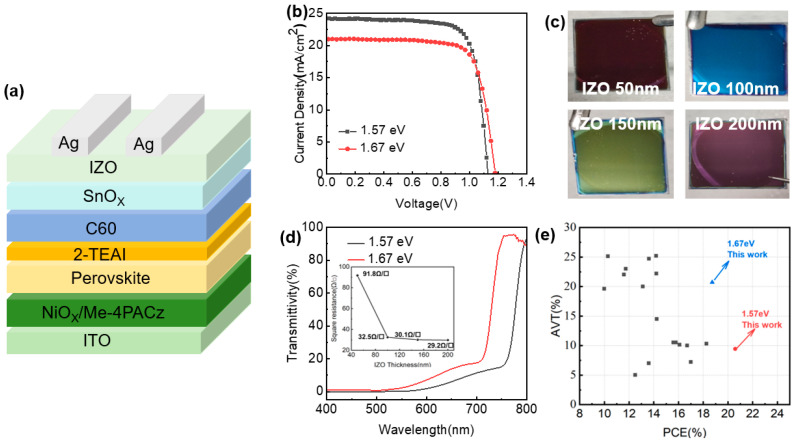
(**a**) Structure of semitransparent PSC devices, (**b**) corresponding JV curves, (**c**) IZO-thickness-dependent photographs, (**d**) optical transmittance of PSCs with 1.57 eV and 1.67 eV perovskite, and (**e**) PCEs and AVTs obtained for semitransparent PSCs in the literatures, the details were listed in Table S6.

Figure 5a shows a diagram of the semitransparent PSCs, in which the sputtered indium zinc oxide (IZO) acted as the transparent electrode and the ALD-deposited SnO_x_ was used as a buffer layer to protect the perovskite films from sputtering damage. Since the wide-bandgap SnO_x_ film is transparent in the visible light region, a thinner IZO is expected to obtain a high AVT for semitransparent devices; however, thin IZO films usually have low conductivity. To address this problem, the square resistance of IZO at various thicknesses (50 nm to 200 nm) was investigated; the corresponding device performance and the AVT are listed in Figure S5 and Table S5. It is remarkable that the 100 nm IZO-related PSCs exhibited the best overall performance; meanwhile, the semitransparent PSCs exhibited a PCE of 20.59% and an AVT of 8.44%. By adding a MoO_x_ optical coupler layer to the IZO cathode, the AVT of PSCs could be increased to 9.45%, as shown in Figure 5d. The JV curves and photographs of the semitransparent PSCs are shown in Figure 5b,c, while the photovoltaic parameters are shown in Table 1; AVTs in the range of 400 nm to 800 nm were calculated from the optical transmittance of semi-PSCs according to a reported method [27]. In addition, the color of PSCs is adjustable by simply changing the IZO thickness, which broadens their applicative potential in BIPV. On the other hand, an AVT of approximately 10% may not meet the requirements of applications that demand high levels of light transmission. Fortunately, the bandgap of perovskite could be adjusted by designing an appropriate precursor component, and a perovskite absorber with a wider bandgap was prepared by bromine ion doping. The resulting FA_0.78_Cs_0.22_PbBr_0.45_I_2.55_ had an optical bandgap of 1.67 eV (Figure S5b), which is higher than that reported previously (1.57 eV). As shown in Figure 5b,d, an optimized AVT of 20.71% and a PCE of 18.73% were obtained for the semitransparent PSC with 1.67 eV perovskite; the corresponding opaque device showed a PCE of 20.26% (Figure S5c). Furthermore, the PCEs and AVTs obtained for semitransparent PSCs in the literature are listed in Figure 5e and Table S6. We believe that, in this study, we have obtained the highest PCE at similar AVTs and the highest AVT at similar PCEs. Consequently, the combination of bifacial modification and the adjustment of the bandgap of perovskite enabled the fabrication of efficient semitransparent solar cells with high AVTs.

## 3. Materials and Methods

### 3.1. Materials

Isopropanol (IPA), N,N-dimethylformamide (DMF), N-methyl-2-pyrollidone (NMP), and chlorobenzene (CB) solvents were purchased from Sigma-Aldrich (Shanghai) Trading Co. (Shanghai, China) Methylammonium iodide (MAI), formamidinium iodide (FAI), cesium bromide (CsBr), cesium iodide (CsI), lead (II) iodide (PbI_2_), lead (II) chloride (PbCl_2_), lead (II) bromide (PbBr_2_), 2-thiopheneethylammonium iodide (2-TEAI), [4-(3,6-Dimethyl-9H-carbazol-9-yl)butyl]phosphonic acid (Me-4PACz), fullerene (C_60_), and NiO_x_ nanoparticles were provided by Xi’an Yuri Solar Co.(Xi’an, China) and Liaoning Youxuan New Energy Technology Co. (Yingkou, China) The IZO target and Ag were purchased from Zhongnuo New Material Technology Co. (Beijing, China) All materials were used as received without further purification.

### 3.2. Device Fabrication

The ITO (~7 Ω/sq)-coated glass substrates were ultrasonically cleaned in Decon-90, IPA, and DI water. The dry substrates were treated using UV ozone for 30 min before use. The NiO_x_ solution (20 mg/mL in DI water) was spin-coated (3000 r/min, 40 s) on the UV-treated ITO substrate and annealed (150 °C, 20 min) to form the hole transport layer. Then, a Me-4PACz/IPA solution (0.3–1 mg/mL) was coated (3000 r/min, 45 s) on NiO_x_ and annealed at 150 °C for 20 min as the buried layer of perovskite. Inorganic and organic precursors of MA_0.7_FA_0.3_PbI_2.55_Cl_0.45_ were prepared by dissolving 67 mg of PbCl_2_ and 627 mg of PbI_2_ in 1 mL of DMF or 140 mg of MAI and 60 mg of FAI in 2 mL of IPA, respectively. The inorganic precursor was first spin-coated (3000 r/min, 45 s) on the UV-treated NiO_x_ surface, and the organic precursor was subsequently coated under the same conditions. The perovskite film was obtained after annealing at 100 °C for 10 min. A 2-TEAI/IPA (1–5 mg/mL) passivator solution was coated (4000 r/min, 30 s) on perovskite and annealed at 100 °C for 10 min. Further, the electron transport layer of C_60_ (30 nm) and the Ag (100 nm) electrode were thermally evaporated on perovskite through a shadow mask. The active area of the opaque PSCs was 0.075 cm^2^. For the semitransparent devices, a 15 nm thick SnO_x_ buffer layer was deposited on the sample of ITO/NiO_X_/Me-4PACz/perovskite/C_60_ via ALD, employing TDMASn and H_2_O sources at 85 °C for ~100 cycles. IZO electrodes with various thicknesses were sputtered (room temperature, radio frequency mode, 45 W) on the SnO_x_ buffer. After the Ag grid line and MoO_x_ (10 nm) optical coupler layer evaporated, semitransparent PSCs were fabricated. For the PSCs with a 1.67 eV perovskite absorber, the processes were generally the same. However, the differences are listed as follows: 187.8 mg of FAI, 80.8 mg of CsI, 500.2 mg of PbI_2_, and 115.6 mg of PbBr_2_ were dissolved in 1 mL of DMF and NMP (mixed at a volume ratio of 4:1) to prepare the precursor of FA_0.78_Cs_0.22_PbBr_0.45_I_2.55_. This precursor was spin-coated (1000 r/min for 5 s and 4000 r/min for 40 s) on the HTL, and 0.2 mL of CB anti-solvent was added in the first 10 s. The FA_0.78_Cs_0.22_PbBr_0.45_I_2.55_ film was formed after annealing at 100 °C for 10 min. Then, MACl (0.3 mg/mL) was used as a passivator for the perovskite, which was thoroughly investigated in our previous report [32].

### 3.3. Characterization

SEM, XRD, XPS, UPS, a UV-visible spectrophotometer, TRPL, and a contact angle meter were used to analyze the properties of the perovskite-related films. A solar simulator, source meter, JV and quantum efficiency measurement systems, TPC, TPV, CV, and electrochemical impedance spectroscopy (EIS) were used to evaluate the photovoltaic performance of the PSCs. Details of these tests and instruments can be found in our previous work [33].

## 4. Conclusions

In summary, we proposed a bifacial modification method for perovskite by introducing a Me-4PACz buried layer and a 2-TEAI surface passivator. This strategy improved the crystallization quality and the light absorption of perovskite, aligned the energy level between the perovskite and the anode/cathode, enhanced carrier extraction and transport, suppressed carrier recombination, reduced non-radiative loss, and created superior p-i-n PSCs with a high PCE of 22.62% (steady-state PCE of 21.77%) and V_OC_ of 1.18 V. This strategy also improved the stability of these PSCs under air and high-temperature conditions. Furthermore, by substituting the opaque Ag with a 100 nm thick IZO transparent electrode, the semitransparent perovskite (1.57 eV) devices obtained a PCE of 20.59% and an AVT of 9.45%, with a PCE of 18.73% and an AVT of 20.71% for the 1.67 eV perovskite device. These semitransparent PSCs with excellent overall performance can, therefore, promote the application of perovskites in BIPV in the future.

## Figures and Tables

**Table 1:** Optimal photovoltaic parameters of opaque and semitransparent PSCs with various modifications.

Device		J_SC_ (mA/cm^2^)	FF (%)	PCE (%)	AVT (%)	V_OC_ (V)
1.57 eV-perovskite	Control	24.16	73.68	19.23	/	1.08
	Me-4PACz	24.48	76.38	21.31	/	1.14
	Me-4PACz & 2-TEAI	24.76	77.43	22.62	/	1.18
	Semitransparent	24.20	76.14	20.59	9.45	1.12
1.67 eV-perovskite	Opaque	21.71	77.80	20.26	/	1.20
	Semitransparent	21.03	75.46	18.73	20.71	1.18

## Data Availability

Data are available upon request.

## Supplementary Materials

The following supporting information can be downloaded at https://www.mdpi.com/article/10.3390/molecules30061237/s1: Figure S1: Tauc plots of the (a) control and (b) bifacially modified perovskite films; Figure S2: XPS core-level spectra of (a) C 1s, (b) S 2p, and (c) N 1s for perovskite films with and without 2-TEAI modification. Before the XPS analysis, the data were calibrated by the standard C1s peak; Figure S3: JV curves of PSCs with modifications at various concentrations of (a) Me-4PACz and (b) 2-TEAI; Figure S4: Normalized PCEs of the control and Me-4PACz and 2-TEAI-modified PSCs (a) in a N_2_-filled glovebox for 1000 h and (b) at 100 °C in air for 5 h; Figure S5: (a) JV curves of semitransparent devices with 1.57 eV perovskite and various IZO thicknesses, (b) Tauc plots of the FA_0.78_Cs_0.22_PbBr_0.45_I_2.55_ perovskite film, (c) JV curves of opaque devices with 1.67 eV perovskite and MACl passivator; Table S1: TRPL fitting parameters of the NiO_x_/perovskite, NiO_x_/Me-4PACz/perovskite, and NiO_x_/Me-4PACz/perovskite/2-TEAI samples; Table S2: Photovoltaic parameters of PSCs with modifications at various concentrations of Me-4PACz and 2-TEAI; Table S3: Trap density calculation from SCLC curves for hole-only devices; Table S4: Fitting parameters of the EIS measurement from Nyquist curves with different conditions; Table S5: Photovoltaic and AVT parameters of semitransparent PSCs with IZO electrodes with various thicknesses; Table S6: Comparison of our work with the recent literature regarding semitransparent PSCs. Refs [34,35,36,37,38,39,40,41,42,43,44,45,46,47,48,49] are cited in Supplementary Materials.
