# Supplementary material for: Fabrication of Bifacial-Modified Perovskites for Efficient Semitransparent Solar Cells with High Average Visible Transmittance
Source: Molecules. 2025 Mar 10;30(6):1237. doi: 10.3390/molecules30061237 (PMC11946856; doi:10.3390/molecules30061237)
Supplement: Supplementary file 1 [file molecules-30-01237-s001.zip › molecules-3504088-supplementary.pdf]

Bifacial modified perovskites toward efficient semitransparent solar cells with the high average visible transmittance

Dazheng Chen*[,a], Wenjing Shi[a], Yan Gao[a], Sai Wang[a], Baichuan Tian[a], Zhizhe Wang[b], Weidong Zhu[a], Long Zhou[a], He Xi[a], Hang Dong[a], Wenming Chai[a], Chunfu Zhang*[,a], Jincheng Zhang[a], Yue Hao[a]

a State Key Laboratory of Wide Bandgap Semiconductor Devices and Integrated Technology, Faculty of Integrated Circuit, Xidian University, Xi'an, Shaanxi, 710071, China

b Science and Technology on Reliability Physics and Application of Electronic Component Laboratory, China electronic product reliability and environmental testing research institute, Guangzhou, 511370, China

*Corresponding author. Dazheng Chen (dzchen@xidian.edu.cn) and Chunfu Zhang (cfzhang@xidian.edu.cn)

Table S1 TRPL fitting parameters of the NiO_x/perovskite, NiO_x/Me-4PACz/perovskite, NiO_x/Me-4PACz/perovskite/2-TEAI samples. The method of double exponential fitting ($f(t)=\sum A_i e^{-t/\tau_i}+B$) can be found in reference [1].

	τ_1 (ns)	τ_2 (ns)	A_1	A_2	τ (ns)
NiO_x/perovskite	4.51	82.15	3.31	0.68	65.88
NiO_x/Me-4PACz/perovskite	14.97	47.08	0.51	0.90	42.19
NiO_x/Me-4PACz/perovskite/2-TEAI	10.73	38.06	0.89	0.81	31.60

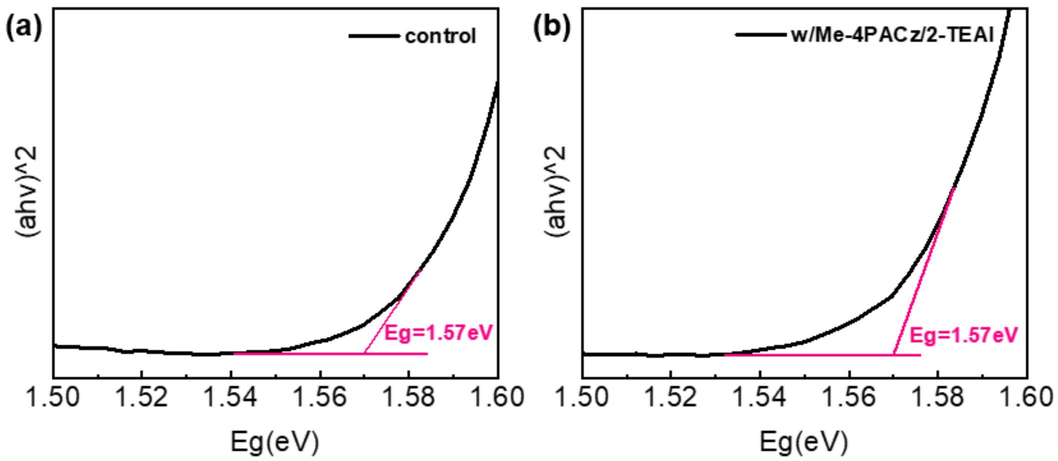

Fig. S1 Tauc plots of the (a) control and (b) bifacially modified perovskite films.

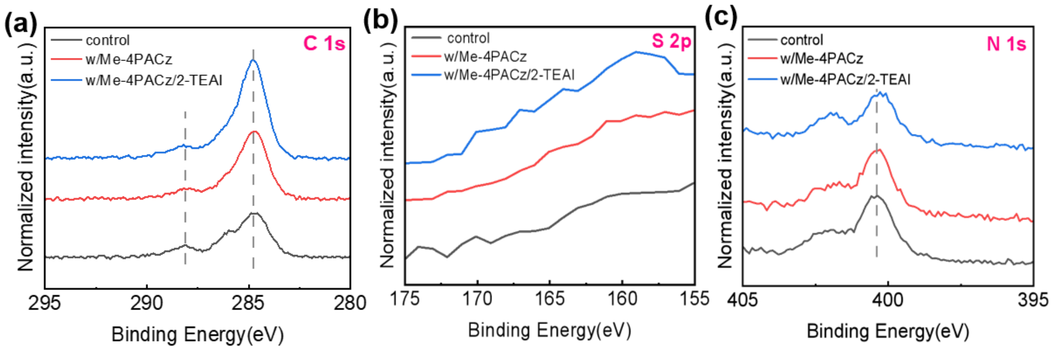

Fig. S2 XPS core-level spectra of (a) C 1s, (b)S 2p, and (c) N 1s for perovskite films with and without 2-TEAI modification. Before the XPS analysis, the data has been calibrated by the standard C1s peak.

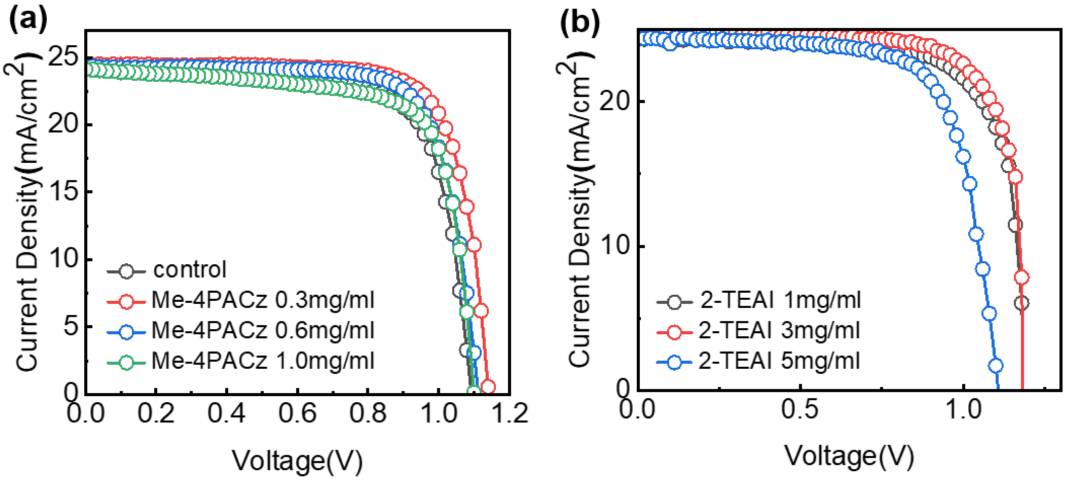

Fig. S3 JV curves of PSCs with modifications at various concentrations of (a) Me-4PACz and (b) 2-TEAI.

Table S2. Photovoltaic parameters of PSCs with modifications at various concentrations of Me-4PACz and 2-TEAI.

Me-4PACz concentration (mg/mL)	V_{OC} (V)	J_{SC} (mA/cm^2)	FF (%)	PCE (%)
0.6	1.10	24.32	76.24	20.39
1.0	1.10	24.10	73.44	19.46
2-TEAI concentration (mg/mL)	V_{OC} (V)	J_{SC} (mA/cm^2)	FF (%)	PCE (%)
1.0	1.18	24.50	75.05	21.69
5.0	1.10	24.47	71.57	19.27

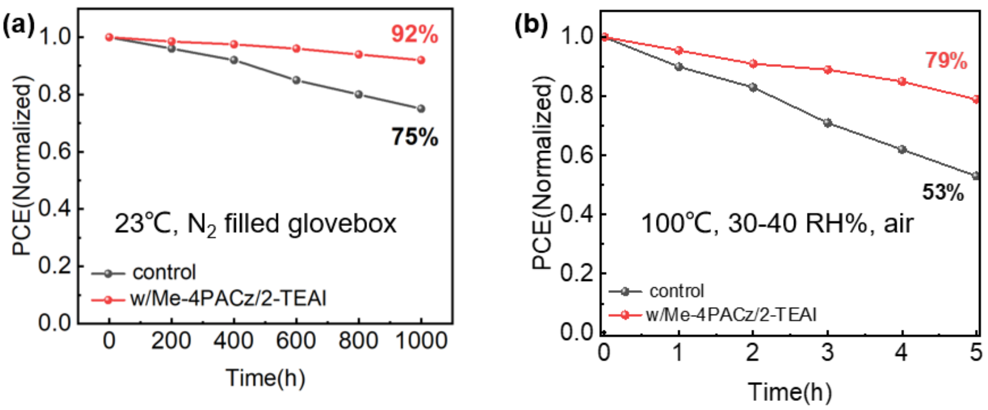

Fig. S4 Normalized PCEs of the control and Me-4PACz & 2-TEAI modified PSCs under (a) N_2 filled glovebox during 1000 hours and (b) 100℃ for 5 hours in air.

Table S3 Fitting parameters of the EIS measurment from Nyquist curves with different conditions. The calculating method can be found in reference [3].

	R_{rec} (Ω)
NiO_x/perovskite	844.7
NiO_x/Me-4PACz/perovskite	1051
NiO_x/Me-4PACz/perovskite/2-TEAI	1754

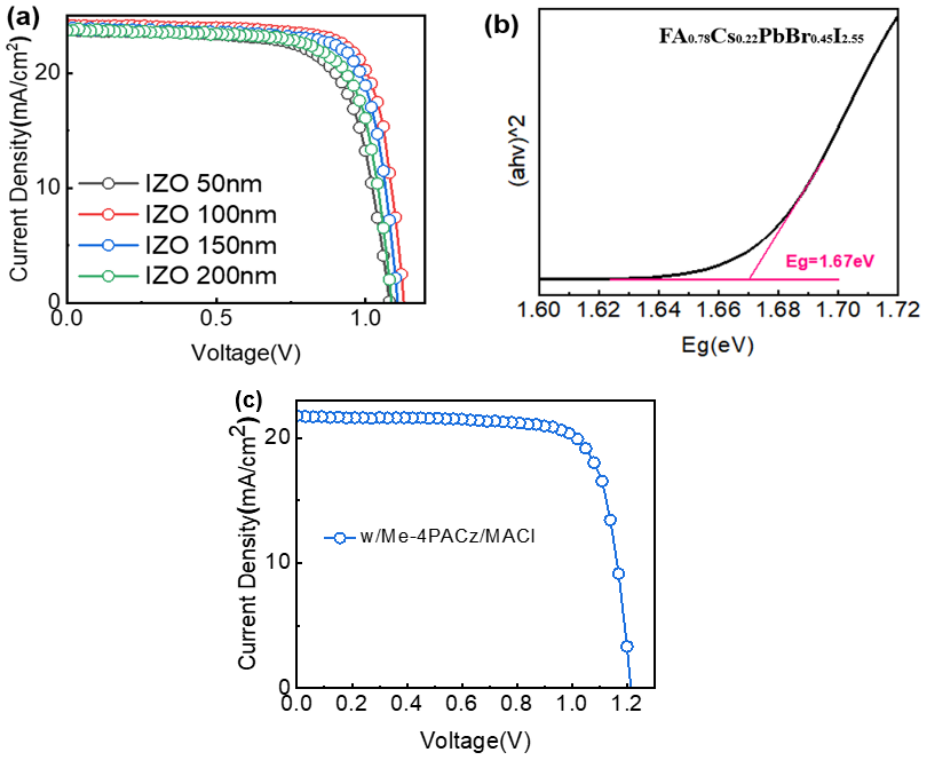

Fig. S5 (a) JV curves of semitransparent devices with 1.57 eV-perovskite and various IZO thicknesses, (b) Tauc plots of the $FA_{0.78}Cs_{0.22}PbBr_{0.45}I_{2.55}$ perovskite film, (c) JV curves of opaque devices with 1.67 eV-perovskite and MACl passivator.

Table S4 Trap density calculation from SCLC curves for hole-only devices. The calculating method ($N_{trap} = 2\varepsilon\varepsilon_0 V_{TFL}/(qL^2)$) can be found in reference [2].

	V_{TFL} (V)	n_{trap} (cm^{-3})
NiO_x/perovskite	0.78	1.34×10^{15}
NiO_x/Me-4PACz/perovskite	0.75	1.28×10^{15}
NiO_x/Me-4PACz/perovskite/2-TEAI	0.70	1.20×10^{15}

Table S5. Photovoltaic and AVT parameters of semitransparent PSCs with various thicknesses of IZO electrodes.

1.57 eV perovskite		V_{OC} (V)	J_{SC} (mA/cm^2)	FF (%)	PCE (%)	AVT (%)
IZO thickness (nm)	Square resistance (Ω/☐)					
50	91.8	1.08	23.85	70.79	18.16	7.33
100	32.5	1.12	24.20	76.14	20.59	8.44
150	30.1	1.10	23.91	76.77	20.19	8.39
200	29.2	1.08	23.83	74.59	19.20	6.80
1.67 eV perovskite with 100 nm IZO		V_{OC} (V)	J_{SC} (mA/cm^2)	FF (%)	PCE (%)	AVT (%)
		1.18	21.03	75.46	18.73	17.53

Table S6 Comparation with recent literature of semitransparent PSCs with our work.

No.	Band gap (eV)	V_{OC} (V)	PCE (%)	AVT (%)	Reference
1	~1.58	1.02	15.8	10.5	4
2	~1.55	0.99	13.6	7.0	5
3	~1.55	1.07	10.3	25.1	6
4	~1.58	1.06	16.10	10.13	7
5	1.59	0.85	17.0	7.20	8
6	1.60	1.05	13.12	20.0	9
7	1.61	0.99	12.50	5.0	10
8	1.60	1.06	11.6	22.0	11
9	1.60	1.03	10	19.6	12
10	1.60	1.06	14.25	14.5	13
11	1.60	1.08	11.74	23.0	14
12	1.62	1.10	18.27	10.3	15
13	1.64	1.08	13.61	24.7	16
14	1.68	1.02	15.60	10.5	17
15	1.70	1.08	16.70	10.0	18
16	1.73	1.26	14.20	25.2	19
17	1.73	1.20	14.21	22.2	20
**18**	**1.57**	**1.12**	**20.59**	**9.45**	**This work**
**19**	**1.67**	**1.18**	**18.73**	**20.71**	**This work**

Reference

[1] Gillespie S C, Gautier J, Van Der Burgt J S, et al. Silicon-Inspired Analysis of Interfacial Recombination in Perovskite Photovoltaics. Advanced Energy Materials, 2024, 14(35): 2400965.

[2] Vincent M. Le Corre, Elisabeth A. Duijnstee, Omar El Tambouli, James M. Ball, Henry J. Snaith, Jongchul Lim, L. Jan Anton Koster, Revealing Charge Carrier Mobility and Defect Densities in Metal Halide Perovskites via Space-Charge-Limited Current Measurements, ACS Energy Lett. 2021, 6, 3, 1087–1094.

[3] Long Zhou, Zhenhua Lin, Zhijun Ning, Tao Li, Xing Guo, Jing Ma, Jie Su, Chunfu Zhang, Jincheng Zhang, Shengzhong Liu, Jingjing Chang, Yue Hao, Highly Efficient and Stable Planar Perovskite Solar Cells with Modulated Diffusion Passivation Toward High Power Conversion Efficiency and Ultrahigh Fill Factor, Sol. RRL, 2020, 3, 1900293.

[4] LIM S H, SEOK H J, CHOI D H, et al. Room temperature processed transparent amorphous InGaTiO cathodes for semi-transparent perovskite solar cells[J]. ACS Applied Materials & Interfaces, 2021, 13(23): 27353-27363

[5] JUNG J W, CHUEH C C, JEN A K Y. High-performance semitransparent perovskite solar cells with 10% power conversion efficiency and 25% average visible transmittance based on transparent CuSCN as the hole-transporting material[J]. Advanced Energy Materials, 2015, 5(17): 1500486.

[6] LIM S H, SEOK H J, KWAK M J, et al. Semi-transparent perovskite solar cells with bidirectional transparent electrodes[J]. Nano Energy, 2021, 82: 105703. https://www.sciencedirect.com/science/article/pii/S2211285520312763

[7] YU J C, SUN J, CHANDRASEKARAN N, et al. Semi-transparent perovskite solar cells with a cross-linked hole transport layer[J]. Nano Energy, 2020, 71: 104635.

[8] Ka, I.; Asuo, I. M.; Basu, S.; Fourmont, P.; Gedamu, D. M.; Pignolet, A.; Cloutier, S. G.; Nechache, R. Hysteresis-Free 1D Network Mixed Halide-Perovskite Semitransparent Solar Cells. Small 2018, 14, 1802319.

[9] Rai, M.; Yuan, Z.; Sadhu, A.; Leow, S. W.; Etgar, L.; Magdassi, S.; Wong, L. H. Multimodal Approach towards Large Area Fully Semitransparent Perovskite Solar

Module. Adv. Energy Mater. 2021, 11, 2102276.
[10] Giuliano, G.; Cataldo, S.; Scopelliti, M.; Principato, F.; Chillura Martino, D.; Fiore, T.; Pignataro, B. Nonprecious Copper-Based Transparent Top Electrode via Seed Layer–Assisted Thermal Evaporation for High-Performance Semitransparent n-i-p Perovskite Solar Cells. Adv. Mater. Technol. 2019, 4, 1800688.
[11] Bisconti, F.; Giuri, A.; Dominici, L.; Carallo, S.; Quadrivi, E.; Po, R.; Biagini, P.; Listorti, A.; Corcione, C. E.; Colella, S.; Rizzo, A. Managing transparency through polymer/perovskite blending: A route toward thermostable and highly efficient, semitransparent solar cells. Nano Energy 2021, 89, 106406.
[12] He, C.; Wang, J.; Chen, S.; Zhou, Y.; Jiang, N.; Zhang, J.; Duan, Y. Resolving the Contradiction between Efficiency and Transparency of Semitransparent Perovskite Solar Cells by Optimizing Dielectric-Metal-Dielectric Transparent Top Electrode. Solar RRL 2023, 7, 2300126.
[13] Cheng, F.; Cao, F.; Chen, B.; Dai, X.; Tang, Z.; Sun, Y.; Yin, J.; Li, J.; Zheng, N.; Wu, B. 85 °C/85%-Stable n-i-p Perovskite Photovoltaics with NiOx Hole Transport Layers Promoted By Perovskite Quantum Dots. Adv. Sci. 2022, 9, 2201573.
[14] Zhang, Y.-W.; Cheng, P.-P.; Tan, W.-Y.; Min, Y. Balance the thickness, transparency and stability of semi-transparent perovskite solar cells by solvent engineering and using a bifunctional additive. Appl. Surf. Sci. 2021, 537, 147908.
[15] Yang, Z.; Niu, Y.; Zhang, X.; Zhang, Z.; Hu, L. Efficiency improvement of semi-transparent perovskite solar cells via crystallinity enhancement. J. Mater. Chem. A 2023, 11, 3070-3079.
[16] Lim, S.-H.; Seok, H.-J.; Kwak, M.-J.; Choi, D.-H.; Kim, S.-K.; Kim, D.-H.; Kim, H.-K. Semi-transparent perovskite solar cells with bidirectional transparent electrodes. Nano Energy 2021, 82, 105703.
[17] Lim, S.-H.; Seok, H.-J.; Choi, D.-H.; Kim, S.-K.; Kim, D.-H.; Kim, H.-K. Room Temperature Processed Transparent Amorphous InGaTiO Cathodes for Semi Transparent Perovskite Solar Cells. ACS Appl. Mater. Interfaces 2021, 13, 27353-27363.
[18] Yu, J. C.; Sun, J.; Chandrasekaran, N.; Dunn, C. J.; Chesman, A. S. R.; Jasieniak,

J. J. Semi-transparent perovskite solar cells with a cross-linked hole transport layer. Nano Energy 2020, 71, 104635.
[19] Yu, J. C.; Li, B.; Dunn, C. J.; Yan, J.; Diroll, B. T.; Chesman, A. S. R.; Jasieniak, J. J. High-Performance and Stable Semi-Transparent Perovskite Solar Cells through Composition Engineering. Adv. Sci. 2022, 9, 2201487.
[20] Rabindranath Garai, Bhavna Sharma, Mohammad Adil Afroz, Shivani Choudhary, Tejasvini Sharma, Isaac Metcalf, Naveen Kumar Tailor, Parameswar K. Iyer, Aditya D. Mohite and Soumitra Satapathi, High-Efficiency Semitransparent Perovskite Solar Cells Enabled by Controlling the Crystallization of Ultrathin Films, ACS Energy Lett. 2024, 9, 6, 2936–2943.
